# Anthocyanins and Functional Compounds Change in a Third-Generation Snacks Prepared Using Extruded Blue Maize, Black Bean, and Chard: An Optimization

**DOI:** 10.3390/antiox10091368

**Published:** 2021-08-27

**Authors:** David Neder-Suárez, Daniel Lardizabal-Gutiérrez, José de Jesús Zazueta-Morales, Carmen Oralia Meléndez-Pizarro, Carlos Iván Delgado-Nieblas, Benjamín Ramírez Wong, Néstor Gutiérrez-Méndez, León Raúl Hernández-Ochoa, Armando Quintero-Ramos

**Affiliations:** 1 Departamento de Investigación y Posgrado, Facultad de Ciencias Químicas, Universidad Autónoma de Chihuahua, Circuito Universitario s/n Campus Universitario 2, Chihuahua 31125, Mexico; dneder@uach.mx (D.N.-S.); cmelende@uach.mx (C.O.M.-P.); ngutierrez@uach.mx (N.G.-M.); lhernandez@uach.mx (L.R.H.-O.); 2 Centro de Investigación en Materiales Avanzados, S. C. Avenida Miguel de Cervantes 120, Complejo Industrial Chihuahua, Chihuahua 31109, Mexico; daniel.lardizabal@cimav.edu.mx; 3 Programa de Posgrado en Ciencia y Tecnología de Alimentos, Facultad de Ciencias Químico Biológicas, Universidad Autónoma de Sinaloa, Ciudad Universitaria, Culiacán 80013, Mexico; zazuetaj@uas.edu.mx (J.d.J.Z.-M.); cidelgadonieblas@uas.edu.mx (C.I.D.-N.); 4 Departamento de Investigación y Posgrado en Alimentos, Universidad de Sonora, Rosales y Blvd. Luis Encinas s/n, Hermosillo 83000, Mexico; bramirez@guaymas.uson.mx

**Keywords:** blue corn, black bean, third-generation snacks, *cyanidin-3-glucoside*, *malvidin-3-glucoside*, *pelargonidin-3-glucoside*, *pelargonidin-3-5-diglucoside*, extrusion-cooking, microwave expansion

## Abstract

The effect of extrusion cooking on bioactive compounds in third-generation snacks (TGSE) and microwave-expanded snacks (MWSE) prepared using black bean, blue maize, and chard (FBCS) was evaluated. FBCS was extruded at different moisture contents (MC; 22.2–35.7%), extrusion temperatures (ET; 102–142 °C), and screw speeds (SP; 96–171 rpm). Total anthocyanin content (TAC), contents of individual anthocyanins, viz., *cyanidin-3-glucoside*, *malvidin-3-glucoside*, *pelargonidin-3-glucoside*, *pelargonidin-3-5-diglucoside*, and *delphinidin-3-glucoside* chloride, total phenolic content (TPC), antioxidant activity (AA), and color parameters were determined. TAC and individual anthocyanin levels increased with the reduction in ET. ET and MC affected the chemical and color properties; increase in ET caused a significant reduction in TPC and AA. Microwave expansion reduced anthocyanin content and AA, and increased TPC. Extrusion under optimal conditions (29% MC, 111 rpm, and 120 °C) generated products with a high retention of functional compounds, with high TAC (41.81%) and TPC (28.23%). Experimental validation of optimized process parameters yielded an average error of 13.73% from the predicted contents of individual anthocyanins. Results suggest that the TGSE of FBCS obtained by combining extrusion and microwave expansion achieved significant retention of bioactive compounds having potential physiological benefits for humans.

## 1. Introduction

Changes in food consumption patterns have led to a growing demand for healthy and easy-to-consume snacks. These snacks, along with satiating hunger and cravings, should provide nutritional and health benefits to the consumer. The food industry has shifted their focus toward incorporating functional food ingredients, using low-cost and simple technologies for preparating healthy snacks. Several snacks that are currently available on the market are prepared with or without expansion, and are mostly based on white corn or other nixtamalized flours. For the development of healthy third-generation snacks, pigmented blue or purple corn and black beans present attractive alternatives. These alternatives are rich in bioactive compounds, such as anthocyanins, polyphenols, flavonols, hydroxycinnamic acids, dihydroxybenzoic, ferulic and chlorogenic acids, flavones, isoflavones, and flavanols. These compounds have been associated with cell protection against oxidative damage and other potential health benefits [1,2,3,4]. Damián-Medina et al. [5] suggest that foods rich in anthocyanins, hydroxycinnamic acids, and isoflavone from blue corn and black beans could be used as alternatives of source of bioactive compounds, and could help in the prevention of metabolic disorders, such as type 2 diabetes mellitus. In the same way, Mojica et al. [6] and Smorowska et al. [7] demonstrated the antidiabetic actions of anthocyanin-rich extracts of black beans and blue corn. Moreover, Herrera-Sotero et al. [8] showed the antiproliferative effects on the breast and prostate cancer cell lines, using extracts of blue corn kernels. Blue corn and black beans, and other novel functional ingredients, such as herbaceous plants (e.g., chard), are rich in minerals, fiber, and phenolic compounds [9,10,11]. These ingredients could be used for production of snacks using extrusion technology. Extrusion technology provides various advantages, including high yields, economic feasibility, no pollution, and oil-free cooking; the short processing time reduces the destruction of heat-sensitive compounds and enables better preservation of the bioactive quality [12,13,14]. Most changes in content polyphenols [6,15], flavonoids [16], anthocyanins [1,2,12], and related bioactive compounds during processing have been attributed to the extrusion temperature, which is enhanced by the combination of moisture content and mechanical stresses that are generated during the extrusion process. The effects of processing on the stability and degradation of these functional compounds that are contained in food matrices made of beans and corn have been previously studied [1,16]. Lately, pigmented corn has received increased attention, due to its use as a major alternative ingredient in common snacking products, such as tortillas, chips, pinole, flour, masa, and tamales [17]. The anthocyanin content in pigmented corn gives these products a unique characteristic, differentiating them from other commercial products. Anthocyanins, in their natural form, are esterified with one or several sugars, or contain acyl group [18,19]. Anthocyanin stability is affected by changes in the pH and temperature, and is a subject of extensive research [20,21,22,23]. The degradation of anthocyanins to simpler molecules leads to changes in the color of food matrices. Recent reports have shown that thermal processing of blue and purple corn food matrices could cause the decomposition of anthocyanin-based glucosides, such as cyanidin, pelargonidin, and peonidin, to simple and acylated anthocyanins in processed corn products [4,20,24,25]. Such changes in the anthocyanin levels alter their antioxidant content and activity [21,23,26]. The effects of extrusion-cooking technologies on the increase, degradation, or changes in individual anthocyanins have been previously reported [12,13]. The thermal degradation of polyphenols [22,27,28] and anthocyanins [26,29] has been associated with complex reactions that result in the formation of Maillard products [4,23,24]. Additionally, the moisture content of the mixtures fed to the extruder could affect the stability of anthocyanins or polyphenols [1,2]. Schmid et al. [15] and Menchaca-Armenta et al. [2] suggest that a high moisture content promotes the polymerization of phenolic compounds, which affects their extractability and antioxidant activity. In contrast, processing under a low moisture content (<15%) generates simpler, more extractable forms of the phenolic compounds, and this could be further enhanced in combination with high shear stresses and temperatures [28,30,31]. These chemical changes are associated with structural changes in materials subjected to extrusion or other thermal processes, thereby increasing the release of bioactive compounds contained in the cell wall [2,6,15,27]. The processing of blue corn, alone or in a mixture with other ingredients, such as beans, by extrusion-cooking, or in combination with other technologies, has been reported [2,30,32,33]. The physical properties of the food matrix are also dependent on shear stress, temperature, and pressure generated in the extruder chamber. These processing conditions result in cell wall disintegration, mainly in the grain pericarp, which is the outer skin layer enriched with bioactive compounds [2,18,19,20], thereby promoting the release or degradation of these compounds. Although there are reports on the use of combinatorial thermal technologies for the processing of blue corn and black bean mixtures for the production of snacks, the addition of chard to these ingredients present an opportunity to produce healthy third-generation snacks. A detailed study, including blue corn, black beans, and chard, to investigate the effects of the combination of extrusion technology with microwave expansion under optimal operating conditions, on the individual anthocyanins, polyphenols, and other antioxidant compounds, has not been performed. Hence, the current study aimed to evaluate the effects of extrusion on the compounds in a mixture of blue corn and black beans at different temperatures, moisture content, and screw speed. These extrusion conditions were also determined to optimize the yield of bioactive components, such as individual anthocyanins, polyphenols, and their color properties in a third-generation snack. 

## 2. Materials and Methods

### 2.1. Raw Material

Black beans (*Phaseolus vulgaris* L.) were obtained from Puebla State, Mexico, blue maize (*Zea mays* L.) from the Tarahumara mountains, Chihuahua State, Mexico, and fresh chard (Beta vulgaris) from the local market of Chihuahua State, Mexico; chard leaves was dehydrated at 50 °C for 6 h in a convection oven, with a final moisture content of 7.01%. 

### 2.2. Preparation for Mixture Extrusion

Black beans, blue maize, and dehydrated chard were individually milled in a hammer mill (Pulvex model 200, Mexico city, Mexico) using a 2-mm sieve, and re-sieved with a 400-µm sieve. After the milling process, the moisture content of blue corn and black bean was 5.27% and 8.12%, respectively. The flour for extrusion process (FBCS) obtained from the mixture of black beans (33%), blue maize (66%), and chard (1%), with an initial moisture content (MC) of 6.63%, was conditioned by adding water to obtain different MC (22.2, 25, 29, 33, and 35.7%). Each moistened mixture was packed in a polyethylene bag for tempering, and stored for 14 h at 4 °C.

### 2.3. Extrusion Cooking

Batches of FBSC conditioned at different adjusted moistures were extruded through a twin-screw extruder (Model #LT32L; Shandong Light M&E, China) with an L/D of 18.5, screw compression ratio of 2:1, and a circular die-nozzle with a length of 19 mm and an output of 2 mm. The FBCS was extruded at different screw speed (SP) (96, 111, 133, 157, and 171 rpm) and extrusion temperature (ET). The extruder barrel was divided into three independent heating zones: the first section of the extrusion chamber was maintained at 80 °C, the temperature of the intermediate zone was varied as 102 °C, 110 °C, 122 °C, 134 °C, or 142 °C, and the end section was maintained at 65 °C. ET was controlled using three independent electrical heaters. Combinations of the different study variables are listed in Table 1. Samples of third-generation snacks (TGSE) were air-dried at 25 °C for 24 h, until an MC of 14.23 ± 0.39% was attained.

### 2.4. Microwave Expansion

Microwave expansion of third-generation snacks was determined, as conducted by Neder-Suarez et al. [34], using a microwave oven (model R-501CW, 1000 W and 2450 Hz; Sharp Corp., Osaka, Japan), with an expansion time of 26 s. 

### 2.5. Preparation of 3G Snack Extracts

Extracts for determining the contents of individual anthocyanins were obtained from (TGSE) and microwave-expanded snacks (MWSE), which were ground, sieved with a 250-µm sieve, and stored in plastic bags at 4 °C until further analyses. Samples (2.0 g) of each treatment were mixed with 20 mL of 90% (*v*/*v*) methanol. This dispersion was agitated for 1 min, and sonicated in an ultrasound bath (Branson 1800; Danbury, CT, USA) for 20 min. The supernatant was centrifuged at 3200× *g* (IEC model CL3-R; Thermo, Waltham, MA, USA) for 15 min at 4 °C. The suspension was evaporated using a Rotavapor R-210 (Buchi Labortechnik AG, Flawil, Switzerland). The fraction enriched with anthocyanins was dissolved using 2.5 mL of 90% (*v*/*v*) methanol. The extracts were filtered through a syringe filter with a 0.45 μm nylon membrane, and stored in amber vials for analysis.

### 2.6. Profiles of Total and Individual Anthocyanins 

The identification of total and individual anthocyanins in the TGSE and MWSE extracts was performed using a UHPLC (Thermo Scientific Dionex Ultimate 3000; Sunnyvale, CA, USA), equipped with a UV-visible detector and a diode array detector, according to the method described by Sánchez-Madrigal et al. [35]. Reversed-phase separation was carried out using a Thermo Scientific Dionex C18 (5 μm, 4.6 × 150 mm) column. Aqueous formic acid 8% (Solvent A) and acidified methanol with formic acid 5% *v*/*v* (Solvent B) were used as elution solvents. The samples were eluted according to the linear gradient for 45 min, starting with 20% to 55% solvent B in 30 min, followed by a washing and reconditioning of the column with 20% solvent B for 45 min. The flow rate was 1.0 mL/min, the column temperature was 30 °C, and the injection volume was 20 μL. Detection was performed at a wavelength of 520 nm. The analyses of chromatogram were performed using Chromeleon v.6.80 software (Thermo Fisher Scientific-Dionex, Walthan, MA, USA). The anthocyanins in the sample were identified by their retention times using standards for *cyanidin-3-glucoside* (*C_3_G*), *malvidin-3-glucoside* (*M_3_G*), *pelargonidin-3-glucoside* (*P_3_G*), and *pelargonidin-3-5-diglucoside* (*P_3-5_DG*), *delphinidin-3-glucoside-chloride,* and calculated as retention percent (*D_3_GR*). Total anthocyanins content (TAC) was determined by collecting the areas for all peaks of the spectrum, quantifying them in terms of cyanidin-3-glucoside equivalents and the change in anthocyanin content after microwave heating (R-TAC) was determined by the difference in TAC between TGSE and MWSE. 

### 2.7. Total Phenolic Content (TPC)

TPC was determined according to the Folin–Ciocalteu colorimetry method followed by Ruiz-Armenta et al. [28], with minor modifications. Extracts were prepared by homogenizing 2 g of sample with 5 mL of acidified methanol solution (methanol and 1 N HCl, 99:1, *v*/*v*). Gallic acid was used to prepare the calibration curve, and deionized water was used as the solvent. The absorbance was measured at 760 nm using a spectrophotometer (Lambda 25 UV/VIS; PerkinElmer, MA, USA). The results were expressed as mg of gallic acid equivalents per 100 g of sample (mg GAE/100 g). TPC was determined in triplicate for each treatment, and the average ± standard deviation was calculated.

### 2.8. Antioxidant Activity (AA)

AA was measured using 2,2-diphenyl-1-picrylhydrazyl (DPPH). Samples (2.5 g) were homogenized with 5 mL acidified methanol, extracted for 30 min in an ultrasonic bath (Branson 1800) at 25 °C, and centrifuged at 3000× *g* (5702 R; Eppendorf, Hamburg, Germany) for 20 min. Moreover, 6-hydroxy-2,5,7,8-tetramethylchromane-2-carboxylic acid (Trolox) was used to obtain the calibration curve. The absorbance was measured at 515 nm using a spectrophotometer (Lambda 25 UV/VIS; PerkinElmer, Waltham, MA, USA). The results were expressed as μmol of Trolox equivalents per 100 g of sample (μmol TE/100 g). AA was determined in triplicate for each treatment, and the average ± standard deviation was calculated.

### 2.9. Color Parameters

The parameters of color a* and b* were measured by tristimulus calorimetry using a Konica Minolta CR-400/410 colorimeter (Minolta Co., Osaka, Japan). Samples for each treatment were milled to a particle size of 60 mesh (250 µm). The samples were placed in Petri dishes with a diameter of 5 cm, and 15 readings were obtained for each treatment, followed by the calculation of average ± standard deviation. Chroma and Hue were calculated using Equations (1) and (2):

Chroma = (a^2^ + b^2^)^1/2^ (1)

Hue = tan^−1^ (b/a) (2)

### 2.10. Experimental Design and Statistical Analysis

A five-level, three-variable, central composite rotatable design was used, resulting in 20 treatments (Table 1), which were analyzed using response surface methodology. The independent variables were: MC (*X*_1_), SP (*X*_2_), and ET (*X*_3_). Significant differences were assumed at *p* < 0.05, and the fitted second-order polynomial equation was obtained by Equation (3):

(3) $$Y_{i}=b_{0}+\overset{3}{\underset{j=1}{\sum }}b_{j}X_{j}+\overset{3}{\underset{J=1}{\sum }}b_{jj}X_{j}^{2}+\overset{2}{\underset{J=1}{\sum }}\overset{3}{\underset{k=j+1}{\sum }}b_{jk}X_{j}X_{k}$$

where *Y_i_* is the response variable for experiment *i*, *X_i_* is the predictor variable, and *b*_0_, *b*_1_, *b*_2_, *b*_11_, *b*_22_, and *b*_12_ are estimated coefficients. Analysis of variance, regression, canonical analyses and optimization of process conditions were performed using Design Expert software v.6.01 (Stat-Ease, Inc., Minneapolis, MN, USA). The critical values of the variables (maximum, minimum and saddle points) were determined using JMP Statistical Discovery software v.11.0 (SAS Institute, Cary, NC, USA). Pearson correlations were performed to relate the independent variables using Minitab software v.17.1.0 (Minitab LLC, State College, PA, USA).

## 3. Results and Discussion

### 3.1. Model Fitting

SP and ET had the most significant effect (*p* < 0.05) on the contents of total and individual anthocyanins and MC and ET for TPC, AA, and color properties in TGSE. Whereas in MWSE, the MC and ET showed more marked effects on TPC, AA, color properties, and in the individual anthocyanins as *P_3_GC*, *M_3_GC,* and *D_3_GR* (Table 2; Table 3). In general terms, the proposed second order model was adequately adjusted for most of the responses for both processes (TGSE and MWSE), satisfactorily predicting the behavior with R^2^ values ranging between 0.73–0.97.

### 3.2. Individual Anthocyanins

**Figure 1:**
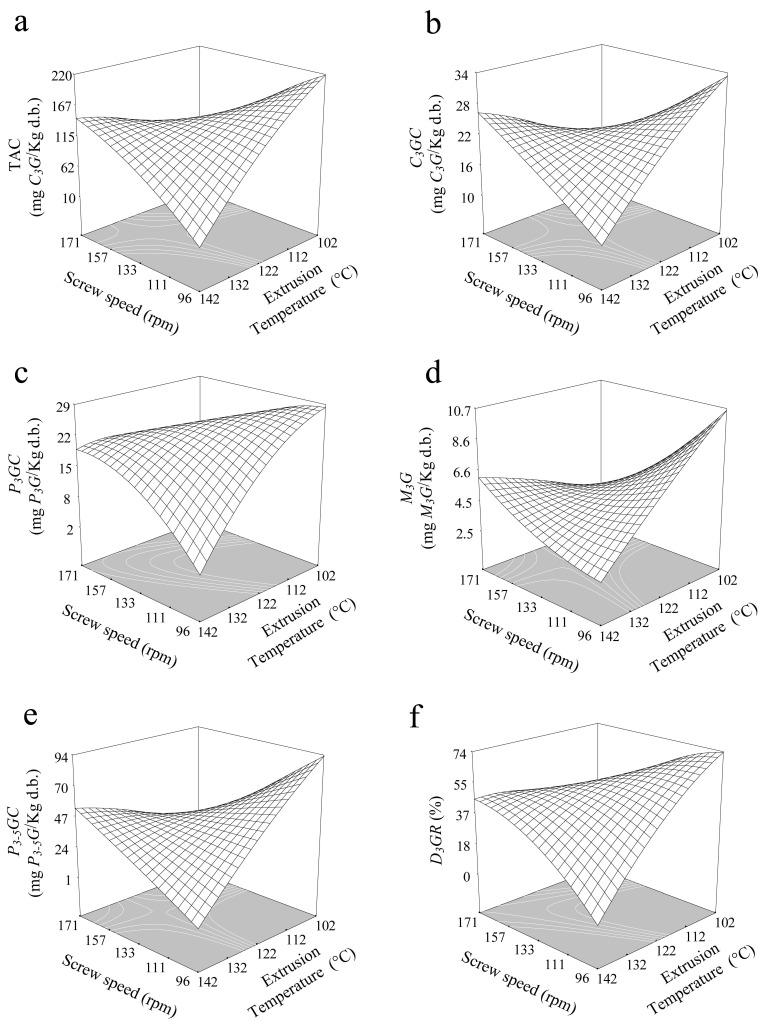
Effect of screw speed and extrusion temperature: (**a**) TAC = Total anthocyanin content, (**b**) *C_3_GC* = *cyanidin-3-glucoside* content, (**c**) P*_3_GC* = *pelargonidin 3-glucoside* content, (**d**) *M _3_GC = malvidin 3-glucoside* content*,* (**e**) *P_3-5_DGC* = *pelargonidin 3-5-diglucoside* content and (**f**) *D_3_GR* = retention percentage of *delphinidin 3-glucoside chloride* in TGSE.

**Figure 2:**
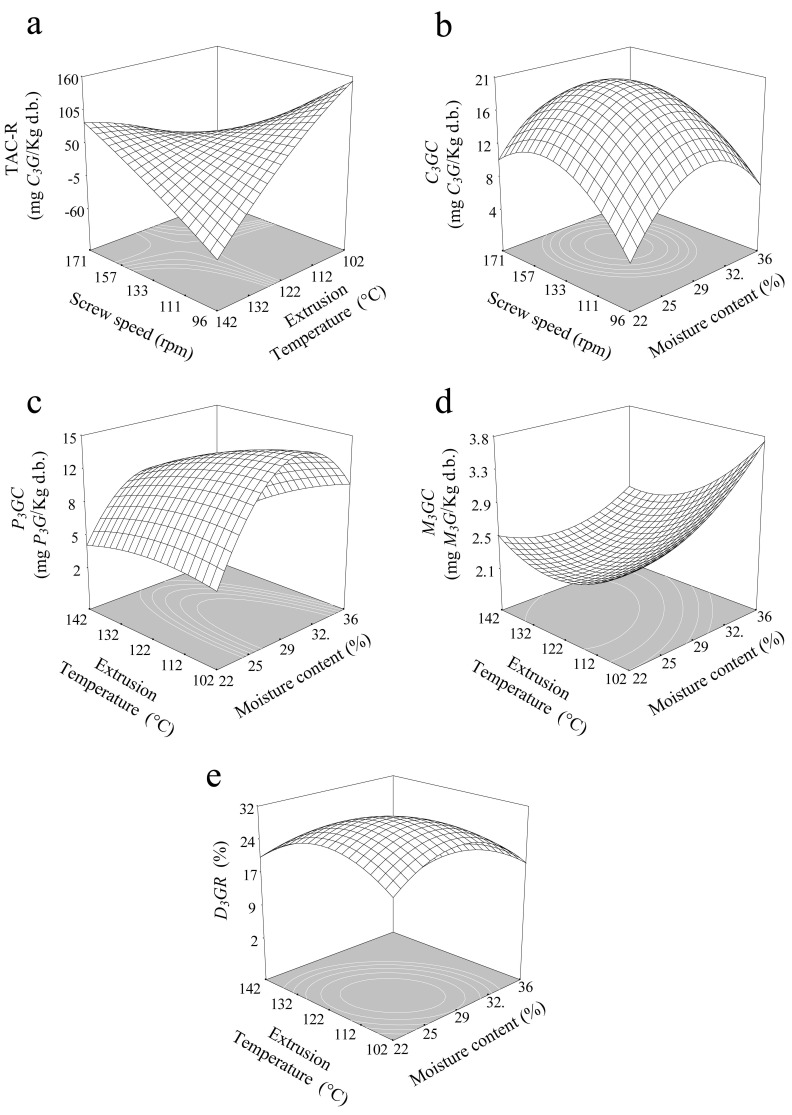
Effect of extrusion temperature, screw speed and moisture content: (**a**) TAC-R = difference of anthocyanin content, (**b**) *C_3_GC* = *cyanidin-3-glucoside* content, (**c**) *P_3_GC* = *pelargonidin 3-glucoside* content, (**d**) *M_3_GC = malvidin 3-glucoside* content and (**e**) *D_3_GR* = retention percentage of *delphinidin 3-glucoside chloride* in MWSE.

Anthocyanins are a major group of phenolic pigments that are responsible for the violet/blue color in cereals, vegetables, roots, and tubers [1,18,19,20,25,26]. Cyanidin glycosides were the major anthocyanidins identified in Mexican blue maize, constituting 71–93% of the total anthocyanins [4,20,24,25,26]. Individual anthocyanins content for blue corn, black beans, and FBCS are shown in Table 4. After extrusion, all treatments resulted in decreased TAC. The decrease in anthocyanin content during processing was due to breaking of covalent bonds, disruption of the cell wall, and decomposition of compounds sensitive to temperature, and the copigment complexes becoming less stable [2,26,27]. SP and ET affected the anthocyanin content significantly (Table 2); the model explains the data variability 86% (R^2^ = 0.86), presenting a saddle point of 131.74 mg *C_3_G*/kg for 29.19%, at 128.79 °C and 148.96 rpm. Under this critical condition, 47% of the initial TAC content, with respect to FBCS, was retained (Table 4). Figure 1a shows that high temperature and low screw speed caused reduction of TAC, whereas low temperature and SP increases the TAC. Similar results were reported by Camacho-Hernandez et al. [12] in third-generation blue corn snacks. Escalante-Aburto et al. [1] found a reduction in anthocyanins by increasing ET in directly expanded blue maize snacks. It was attributed to the damage caused by the sheer force and heat during extrusion, causing a reduction in anthocyanin content [1,14]. After microwave heating, a reduction in total anthocyanin (R-TAC) was generated. R-TAC was affected by the interaction of SE and ET; the adjusted second-order model presented a behavior-type saddle, with a value of 56.71 mg *C_3_G*/kg for 30.26% MC, at 125 °C, and 142.80 rpm. Increments in temperature and SP produced greater reduction in TAC (Figure 2a). This result was attributed to the degradation of anthocyanin caused by temperatures under a microwave intensity and low moisture content [29], which could cause the decomposition of anthocyanin-based glucosides to simple and acylated anthocyanins [4,20,24,25]. Total anthocyanin content showed a positive correlation with individual anthocyanins; *C_3_GC* (r = 0.84, *p* < 0.01), *P_3_GC* (r = 0.88, *p* < 0.01), *P_3-5_DGC* (r = 0.84, *p* < 0.01), *M_3_GC* (r = 0.84, *p* < 0.01), and *D_3_GR* (r = 0.93, *p* < 0.01). 

Cyanidin-3-glucoside content in TGSE was significantly affected (*p* < 0.05) by SP and ET. The average *C_3_GC* values ranged from 26.61–16.07 mg/kg. The adjusted model shows a saddle point at 22.20 mg *C_3_G*/kg after extrusion under 27.38% MC, at 136.99 °C and 132.76 rpm, which was which retained 52% of the initial content present in FBCS (Table 4). *C_3_GC* values decreased with increasing ET at low SP, whereas decreasing ET values increased (Figure 1b). Similar findings were reported by Escalante-Aburto et al. [1] for *C_3_GC* in second-generation snacks made using whole blue maize. Some reports [1,12,36] have shown that an increase in temperature generates degradation reactions, causing a reduction in anthocyanin content. After microwave expansion, *C_3_GC* was significantly affected by a quadratic effect of MC and SP. Successive increment or reduction in SP and MC reduced *C_3_GC* (Figure 2b). This bioactive component is temperature sensitive; therefore, during microwave expansion, it was degraded [16,27,28,37,38,39]. The adjusted second-order model for *C_3_GC* exhibited a behavior-type saddle point of 19.45 mg *C_3_G*/kg at 30.56% MC, at 73.07 °C and 148.38 rpm. 

Pelargonidin-3-glucoside content presented similar trend as *C_3_GC* (Figure 1c). The adjusted model shows a saddle point of 3.68 mg *P_3_G*/kg in TGSE. On the other hand, after microwave expansion, *P_3_GC* was significantly affected by ET and MC. Intermediate MC values and low temperatures increases the *P_3_GC*, while high temperatures and low moisture caused a reduction (Figure 2c). MC increases reduce the degradation of anthocyanins, because water acts as a lubricant during extrusion [27,30,33]. The prediction model shows a saddle behavior with a value of 11.94 mg *P_3_G*/kg at 153.08 °C, 29.83% MC, and 62.23 rpm. 

Regarding changes in TGSE for malvidin-3-glucoside content, it was observed that the same behavior of *C_3_GC* and *P_3_GC* was affected by the ET and SP (Figure 1d), resulting in values of 4.73 mg *M_3_G*/kg at 28.19% MC, 125.86 °C, and an extrusion speed of 147.17 rpm. *M_3_GC* showed, in microwave expansion, a different behavior to *C_3_GC* and *P_3_GC*, observing the significative effect by MC and ET. Figure 2d shows that increment in MC at low ET caused an increase in *M_3_GC*. The adjusted model reached a minimum value of 1.90 mg *M_3_G*/kg at 36.12% MC, 140.77 °C and 137.06 rpm. 

Another anthocyanin found was *pelargonidin-3-5 diglucoside*, which was significantly (*p* < 0.05) affected by temperature and SP during extrusion. *P_3-5_DGC* showed a similar trend to previous individual anthocyanins discussed for TGSE (Figure 1e). The trend resulted in a saddle point reaching a value of 43.64 mg *P_3-5_DG*/kg at 30.16% MC, 125.84 °C, and 144.99 rpm. Regarding to microwave expansion in *P_3-5_DGC*, it was not significant (*p* > 0.05).

Delphinidin-3-glucoside-chloride is the major anthocyanin in black beans [39,40,41], and was calculated as retention percent (*D_3_GR*); it ranged between 27.74 and 58.30% and 14.67 and 35.26% for TGSE and MWSE, respectively. An increase in ET decreased *D_3_GR* at low SP, whereas a high SP increased *D_3_GR* slightly (Figure 1f), reaching major values (46.40% retention) for 28.98% MC, at 140.69 °C and 173.96 rpm. Escalante-Aburto et al. [1], Yue and Xu. [38], and Ti et al. [42] demonstrated the degradation or decomposition of anthocyanins during thermal processing. The variables of MC and ET significantly affected the *D_3_GR* during microwave expansion, with resulting values of 31.80% retention, at 113.58 °C, 158.64 rpm, and 26.40% MC. Figure 2e shows that increases or decreases in MC and TE cause a reduction in this anthocyanin.

### 3.3. Total Phenolic Content (TPC) and Antioxidant Activity (AA)

**Figure 3:**
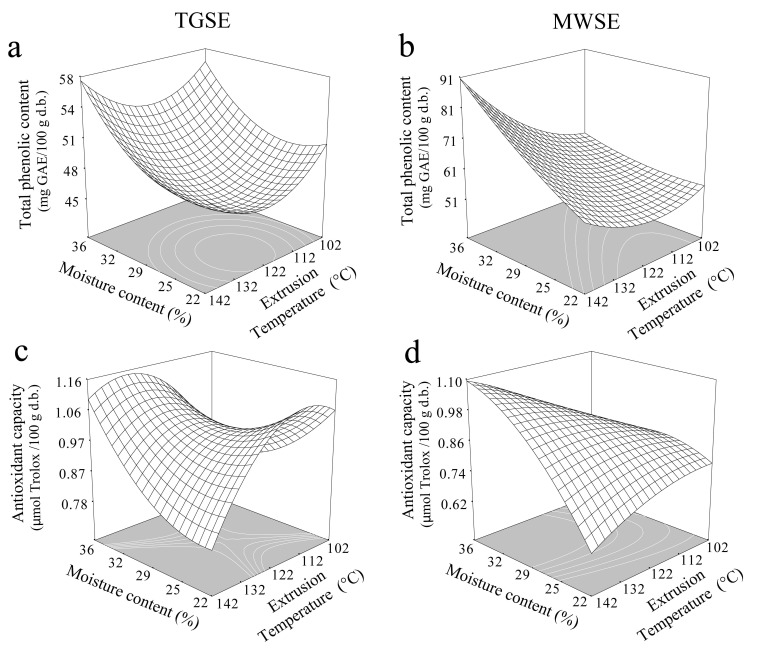
The effect of extrusion temperature and moisture content of total phenolic and antioxidant activity at a screw speed of 133 rpm: (**a**) Total phenolic content of TGSE, (**b**) Total phenolic content of MWSE, (**c**) Antioxidant activity of TGSE, and (**d**) antioxidant activity on MWSE.

TPC and AA of blue corn, black beans, and FBCS are shown in Table 4. After extrusion, the values decreased, ranging between 43.08 and 54.12 mg GAE/100 g. The effects of ET and MC on TPC for TGSE and MWSE are shown in Figure 3a,b, respectively. The fitted model presented significant adjustments, explaining 76% of the variability of the data (R^2^ = 0.76), reaching a minimum value of 42.67 mg GAE/100 g for 20.67% MC, with extrusion at 109.23 °C and 193.80 rpm, under this critical condition, TPC preserved 29% compared to FBCS (Table 4). An increase in ET and reduction in MC increased the TPC in TGSE. Similar findings were reported by Delgado-Nieblas et al. [27], Leyva-Corral et al. [31], Ruiz-Armenta et al. [28], and Zhang et al. [16], which indicates that this reduction may be due to structural damage by feed MC and barrel temperature. Further, TPC was significantly affected by MC and ET following microwave expansion (Table 2). The proposed model adequately estimated the variability in data predicted to be 80% (R^2^ = 0.80), with a saddle point of 50.64 mg GAE/100 g at 18.49% MC, with extrusion at 118.40 °C and 164.00 rpm. High temperatures, along with an increase in MC, caused an increase in TPC (Figure 3b). This increase in TPC may be due to a higher extractability of phenolic compounds and an increase in free and total phenolic compounds of the cell wall. This release of phenolic compounds was stimulated by heating in combination with high shear stresses [15,27,28,31]. All processing steps decreased the AA in the extruded samples compared with FBCS. This reduction of phenolic content and antioxidant activity in extrudates is due to decarboxylation and de-esterification reactions caused by high barrel temperature and moisture content, this may promote the transformation of phenolic acid and conjugated forms into other kinds, leading to reduced extractability and antioxidant activity [6,31]. Authors, such as White et al. [43], suggest that a change in the composition of the phenolic compounds by extrusion cooking is due to breaking covalent bonds in phenolic compounds. In addition, other reducing compounds could be from the Maillard reaction, which occurs during the thermal process; these reactions also increase with temperature [15,22,23,26,30]. Reduction in temperature at low MC, and an increase in MC at high temperatures caused an increase in AA (Figure 3c). AA of TGSE showed a quadratic relation with MC and ET (Table 2). The adjusted model shows a saddle point of 1.175 μmol TE/g at 29.33% MC, for extrusion at 120.12 °C and 139.78 rpm, retaining 37% of the initial content (Table 4). AA at different moisture levels and ETs in MWSE is illustrated in Figure 3d. Analysis of variance indicated that changes in this parameter were affected by MC and ET (Table 3), and the adjusted model yielded a saddle point of 0.90 μmol TE/g at 18.28% MC, 69.81 °C, and 79.35 rpm, which explains 90% (R^2^ = 0.90), of the variability in the data. AA showed a similar trend as TPC following microwave expansion. Extruded samples with high MC exhibited increased AA, whereas those processed at high temperatures and low MC showed a reduction in the same. Similar findings were reported by Ruiz-Armenta et al. [28] and Xu and Chang. [39].

### 3.4. Color Parameters

**Figure 4:**
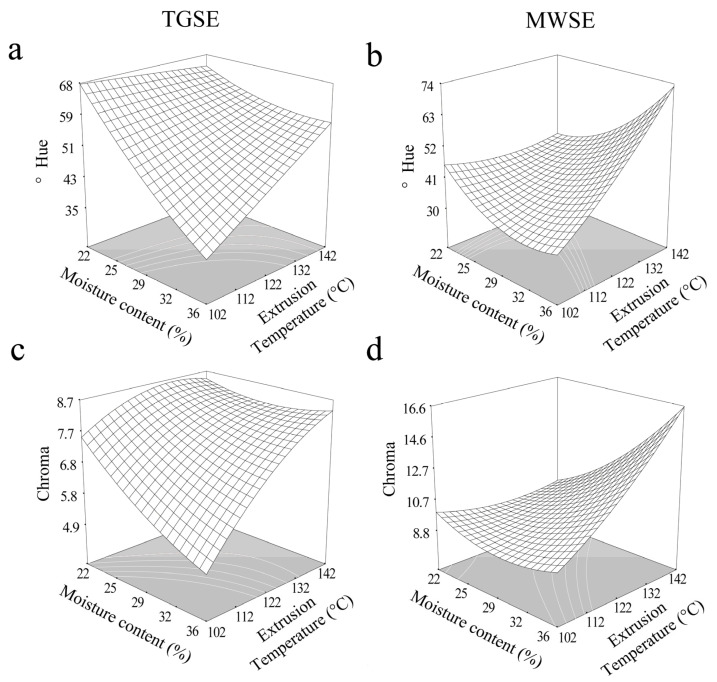
Effect of extrusion temperature and moisture content on color parameters at a screw speed of 133 rpm. (**a**) °Hue of TGSE, (**b**) °Hue of MWSE, (**c**) Chroma of TGSE, and (**d**) Chroma of MWSE.

Color is one of the most important attributes affecting the acceptability of snacks. The extruded products are darker and less reddish, possibly due to Maillard reaction products [13]. The Hue angles obtained for blue corn, black beans, and FBCS are shown in Table 4, indicating red magenta, yellow, and warm red coloration, respectively. MC and ET affected Hue angle significantly (*p* < 0.05) in a linear manner during extrusion, showing a linear decrease with increases in MC, and an increased lineal trend with temperature (Figure 4a). The Hue angles of the extrudates ranged between 40.06 and 64.84°. The adjusted second-order model showed a saddle point, which explains 93% (R^2^ = 0.93) of the variability in the data, with a value of 25.76°. An increase in the Hue angle indicates color degradation, since the blue/purple color is associated with the anthocyanin content and antioxidant activity [18,19,29]. These results suggest the destruction of anthocyanins due to high-processing temperatures. Studies conducted by Escalante-Aburto et al. [1], Camacho-Hernandez et al. [12], and Pozo-Insfran et al. [44] demonstrated that the loss of color is related to a decrease in anthocyanin content after extrusion. Another important measure is Chroma, which indicates color saturation. MC and ET significantly (*p* < 0.05) affected Chroma in TGSE, observing a linear behavior similar to that presented by the Hue parameter (Figure 4c). The adjusted model yielded a saddle point of 8.56 at 24.70% MC, 148.30 °C, and 172.90 rpm. This behavior is due to the heat treatment of anthocyanins, which induces marked structural changes in addition to the loss of their characteristic red-purple color [28,29]. Regarding microwave expansion, the Hue angle was affected by MC and ET in their linear and quadratic effects, the fitted model reaching a saddle point at 108.92°, which explains 96% (R^2^ = 0.96), of the variability in data. An increase in ET and reduction in MC increased the Hue angle (Figure 4b). Decreases in the hue angle are related to the temperature generated during the microwave expansion process. Pozo-Insfran et al. [44] reported an increase in the Hue angle after thermal processing in chips prepared from blue maize tortillas, suggesting the destruction of anthocyanins due to high processing temperatures. Similar results were found by Camacho-Hernández et al. [12] and Escalante-Aburto et al. [31], who showed the degradation of blue corn pigments due to heat treatment, and the loss of their characteristic color. Moreover, Chroma was significantly affected by MC and ET. The model predicted a saddle point of 9.22 at 23.81% MC, 120.40 °C, and 121.90 rpm. Chroma showed a similar trend to the Hue angle in WMSE (Figure 4d). This research seeks to preserve the blue–purple color of the raw material, which is correlated with anthocyanin content and AA. Extrusion processing improves the retention of these pigments when carried out at temperatures below 100 °C and at high MC.

### 3.5. Optimal Conditions

**Figure 5:**
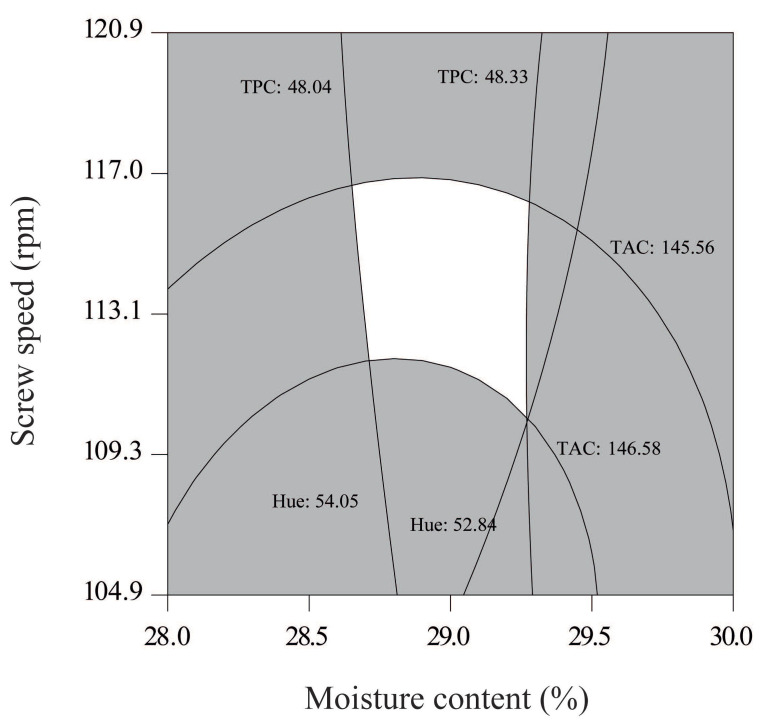
Optimized region obtained of contour plots of the physicochemical characteristics of extruded pellets: TAC = total anthocyanin content (mg *cyanidin 3-glucoside*/kg), TPC = total phenolic content (mg GAE/100 g) and Hue.

Canonical analysis was applied to locate the stationary points for TAC, TPC, and Hue responses. The criterion for graphical optimization was to find the conditions that, after the extrusion, allowed for the highest TAC and TPC, and the lowest Hue. The region that satisfies this criterion served as the basis for identifying the optimal conditions for the process. The overlaid contour plots of the individual TAC, TPC, and Hue plots resulted in an optimal region (Figure 5). The predicted optimum conditions were as follows: MC, 28.63–29.26%; SP, 109.68–116.92 rpm; and ET, 120 °C. 

### 3.6. Process Validation

The optimal conditions for the process were verified experimentally at 29% MC, 111 rpm, and extrusion at 120 °C, and the values for TAC, *C_3_GC*, *P_3_GC*, *P_3-5_DGC*, *M_3_GC*, *D_3_GR*, TPC, and hue were recorded. The overlay plot yielded values for TAC, C3GC, TPC, and hue as 146.69 mg *C_3_G*/kg, 23.60 mg *C_3_G*/kg, 48.17 mg GAE/100 g, and 53.32°, whereas the estimated values were 160.1 ± 3.06 mg C3G/kg, 27.48 ± 0.86 mg C3G/kg, 43.27 ± 0.52 mg GAE/100 g, and 46.84 ± 1.14°, respectively. The errors between the experimentally obtained values and the values estimated by the model were 9.35%, 16.48%, 12.32%, 15.19%, 14.23%, 10.43%, 10.67%, and 12.83% for TAC, *C_3_GC*, *P_3_GC*, *P_3-5_DGC*, *M_3_GC*, *D_3_GR*, TPC, and Hue, respectively (Table 5). Considering the percentage of relative error average (13.73%) of these responses, the adjusted model in the optimal conditions in TGSE can be considered acceptable, considering that the proposed model for different response variables predicts complex quadratic behaviors that are influenced by different sources of variation, such as process temperature, moisture content, and screw speed, among others. 

## 4. Conclusions

The combination of extrusion cooking and microwave heating is an alternative method of producing TGSE, using a mixture of blue maize and black beans, with the highest phytochemical content. Despite the thermal processes, retention of bioactive compounds was high under the optimal process conditions of 29% MC, 120 °C, and 111 rpm, achieving a retention of 41.81% and 28.23% for TAC and TPC, respectively. The highest retention percentages of individual anthocyanins, viz., *C_3_GC*, *D_3_GR*, *P_3_GC*, *M_3_GC*, and *P_3-5_DGC*, were 63.17%, 60.03%, 59.98%, 50.63%, and 42.47%, respectively. A variability of 13.73% between the experimental values and those estimated by the model was observed. The proposed model was adjusted for the analyzed variables. SP and ET showed significant effects on the content of individual anthocyanins in TGSE, whereas MC, ET, and their interactions had the largest effects on individual anthocyanins in MWSE. The chemical and color properties were affected by MC and ET in TGSE and MWSE. These results indicate that it is possible to produce snacks from black beans, blue maize, and chard, while retaining the nutraceutical compounds and potential health benefits, using a combination of extrusion cooking and microwave heating.

## Figures and Tables

**Table 1:** Process variables and levels used in the experimental design.

	Coded Levels			Process Variables		
Treatment	X_1_	X_2_	X_3_	MC (%)	SP (rpm)	ET (°C)
1	−1	−1	−1	25	111	110
2	1	−1	−1	33	111	110
3	−1	1	−1	25	157	110
4	1	1	−1	33	157	110
5	−1	−1	1	25	111	134
6	1	−1	1	33	111	134
7	−1	1	1	25	157	134
8	1	1	1	33	157	134
9	−1.68	0	0	22.2	133	122
10	1.68	0	0	35.7	133	122
11	0	−1.68	0	29	96	122
12	0	1.68	0	29	171	122
13	0	0	−1.68	29	133	102
14	0	0	1.68	29	133	142
15	0	0	0	29	133	122
16	0	0	0	29	133	122
17	0	0	0	29	133	122
18	0	0	0	29	133	122
19	0	0	0	29	133	122
20	0	0	0	29	133	122

X_1_ = MC, X_2_ = SP, X_3_ = ET, MC = moisture content, SP = screw speed and ET = extrusion temperature.

**Table 2:** Regression coefficients and analysis of variance for chemical properties of TGSE.

Coefficient	TGSE									
	TAC	*C_3_GC*	*P_3_GC*	*P_3-5_DGC*	*M_3_GC*	*D_3_GR*	TPC	AA	Hue	Chroma
Intercept	137.39	22.23	52.68	45.66	4.96	52.68	45.91	1.17	54.22	7.57
*b*_1_	0.63	−0.65	−0.81	−0.88	−0.20	−0.81	1.85 **	< 0.01	−5.62 **	−0.38 **
*b*_2_	−8.32 *	−0.92	−2.41	−4.14	−0.53 **	−2.41	−0.99	< 0.01	−0.02	0.11
*b*_3_	−9.83 *	−0.59	−5.29 *	−5.26 *	−0.51 **	−5.29 **	−0.02	−0.01	2.95 **	0.64 **
*b*_12_	2.47	−0.15	1.59	3.31	0.08	1.59	1.39	−0.02	−0.77	0.06
*b*_13_	−1.73	−1.11	−1.32	2.76	0.04	−1.32	0.21	0.05 *	2.20 *	0.22
*b*_23_	26.06 **	3.16 **	8.34 **	11.10 **	0.95 **	8.34 **	1.34	−0.03	1.83	0.21
*b*_11_	−13.55 **	−1.23 *	−7.18 *	−2.87	−0.32 **	−7.18 **	1.28 *	0.08 *	0.75	0.09
*b*_22_	−4.81	−0.14	−2.96	−0.37	0.20	−2.96	1.00	−0.01	−0.79	−0.15
*b*_33_	−6.94	−0.60	−3.09	−1.80	−0.08	−3.09	1.46 *	−0.05 **	−0.01	−0.18
*p*−Value (model)	<0.01	0.01	<0.01	0.04	<0.01	<0.01	0.02	0.04	<0.01	<0.01
Lack to fit	0.01	0.65	0.19	0.11	0.73	0.48	0.56	0.58	0.10	0.07
CV	10.94	20.89	19.69	19.59	9.23	14.17	4.40	5.78	3.87	5.40
R^2^	0.86	0.79	0.83	0.73	0.89	0.83	0.76	0.74	0.93	0.85

* Significance (*p* ˂ 0.05), ** significance (*p* ˂ 0.01); *b*_1_ = moisture content, *b*_2_ = screw speed, *b*_3_ = extrusion temperature, CV = coefficient of variation, TGSE = third-generation snacks, MWSE = microwave-expanded snacks, TAC= total anthocyanin content, *C_3_GC* = *cyanidin-3-glucoside* content*, P_3_GC* = *pelargonidin-3-glucoside* content, *P_3-5_DGC* = *pelargonidin-3-5 diglucoside* content *M_3_GC* = malvidin*-3-glucoside* content, *D_3_GR* = retention percentage of *delphinidin-3-glucoside chloride*, TPC = total phenolic content and AA = antioxidant activity.

**Table 3:** Regression coefficients and analysis of variance for chemical properties of MWSE.

Coefficient	MWSE								
	TAC−R	*C_3_GC*	*P_3_GC*	*M_3_GC*	*D_3_GR*	TPC	AA	Hue	Chroma
Intercept	58.99	20.05	13.26	2.30	29.85	57.94	1.07	38.90	9.92
*b*_1_	3.10	0.12	0.53	−0.16	−2.94 **	5.26 **	0.86 **	2.24 **	0.93 **
*b*_2_	−7.71	0.56	0.11	−0.31 *	0.07	0.47	−0.01	< 0.01	0.13
*b*_3_	−7.68	0.39	−0.27 *	−0.29 *	−2.72 **	4.69 **	0.02 *	5.79 **	0.98 **
*b*_12_	3.14	−0.36	−0.63	−0.08	−1.01	−0.42	0.01	−0.62	0.17
*b*_13_	1.44	−0.46	−0.61	−0.33 *	−1.35	2.35	0.05 **	3.69 **	0.66 **
*b*_23_	26.89 **	−0.40	−0.98	0.22	1.22	2.09	0.01	0.64	0.16
*b*_11_	−8.00	−2.25 **	−2.22 **	0.19	−2.39 *	0.76	−0.03 **	2.31 **	0.29 **
*b*_22_	−1.68	−1.57 **	−0.41	0.35 **	−0.72	−0.35	−0.02	−0.70	−0.10
*b*_33_	−6.23	<−0.01	−0.33	0.27 *	−2.28 *	2.22	−0.01	0.98 *	0.20 *
*p*−Value (model)	0.01	0.01	0.02	0.01	0.01	0.01	<0.01	<0.01	<0.01
Lack to fit	0.06	0.11	0.43	<0.01	0.57	<0.01	0.03	0.16	0.02
CV	30.67	9.72	15.68	14.07	11.99	7.54	4.94	3.91	3.03
R^2^	0.80	0.79	0.78	0.81	0.80	0.80	0.90	0.96	0.97

* Significance (*p* ˂ 0.05), ** significance (*p* ˂ 0.01); *b*_1_ = moisture content, *b*_2_ = screw speed, *b*_3_ = extrusion temperature, CV = coefficient of variation, MWSE = microwave-expanded snacks, TAC-R= difference of anthocyanin content, *C_3_GC* = *cyanidin-3-glucoside* content*, P_3_GC* = *pelargonidin-3-glucoside* content, *M_3_GC* = *malvidin-3-glucoside* content, *D_3_GR* = retention percentage of *delphinidin-3-glucoside chloride*, TPC = total phenolic content and AA = antioxidant activity.

**Table 4:** Physicochemical properties of raw material.

Material	TAC	*C_3_GC*	*P_3_GC*	*M_3_GC*	*P_3-5_DGC*	TPC	AA	Hue
Blue corn	357.92 ± 12.48	77.72 ± 1.17	11.47 ± 0.61	21.85 ± 0.41	nd	126.49 ± 1.35	2.74 ± 0.04	338.8 ± 0.15°
Black bean	239.78 ± 4.11	16.44 ± 1.74	67.74 ± 1.62	7.45 ± 0.27	62.64 ± 0.84	68.32 ± 0.66	2.63 ± 0.01	85.12 ± 0.31°
Chard	nq	nq	nq	nq	nq	1979.01 ± 33.86	12.88 ± 0.09	16.42 ± 0.78°
FBCS	310.90 ± 7.16	43.50 ± 1.56	35.46 ± 0.96	13.32 ± 0.54	46.50 ± 1.31	149.73 ± 0.33	3.25 ± 0.01	290.78 ± 0.14°

Values are the average of duplicated measurements ± standard deviation. TAC = total anthocyanin content (mg *cyanidin 3-glucoside*/kg), *C_3_GC* = *cyanidin-3-glucoside* content (mg *cyanidin 3-glucoside*/kg), *P_3_G* = *pelargonidin 3-glucoside* content (mg *pelargonidin 3-glucoside*/kg), *P_3-5_DGC* = *pelargonidin 3-5 diglucoside* content (mg *pelargonidin 3-5-diglucoside*/kg), *M_3_GC* = *malvidin 3-glucoside* content (mg *malvidin 3-glucoside*/kg), TPC = Total phenolic content (mg GAE/100 g) and AA= Antioxidant activity (mg GAE/100 g). nd = not detected, nq = not quantified.

**Table 5:** Experimental and predicted values of the responses variables.

	Experimental	Estimated	% Error
*TAC*	160.41 ± 3.06	146.69	9.35
*C_3_GC*	27.48 ± 0.86	23.60	16.48
*P_3_GC*	21.27 ± 0.36	24.26	12.32
*P_3-5_DGC*	59.95 ± 0.77	52.04	15.19
*M_3_GC*	6.74 ± 0.89	5.90	14.23
*D_3_GR*	60.03 ± 1.36	54.36	10.43
TPC	43.27 ± 0.52	48.17	10.67
Hue	46.84 ± 1.14	53.32	12.83

Values presented are the average of duplicated of the experiment ± standard deviation. TAC = total anthocyanin content (mg *cyanidin 3-glucoside*/kg), *C_3_GC* = *cyanidin-3-glucoside* content (mg *cyanidin 3-glucoside*/kg), *P_3_G* = *pelargonidin 3-glucoside* content (mg *pelargonidin 3-glucoside*/kg), *P_3-5_DGC* = *pelargonidin 3-5 diglucoside* content (mg *pelargonidin 3-5-diglucoside*/kg), *M_3_GC* = malvidin 3-glucoside content (mg *malvidin 3-glucoside*/kg), *D_3_GR* = retention percentage of *Delphinidin 3-glucoside* and TPC = total phenolic content (mg GAE/100 g).

## Data Availability

Data are contained within the article.

## References

[1] Escalante-Aburto A., Ponce-García N., Ramírez-Wong B., Torres-Chávez P.I., Figueroa-Cárdenas J.D.D., Dorado R.G. (2016). Specific Anthocyanin Contents of Whole Blue Maize Second-Generation Snacks: An Evaluation Using Response Surface Methodology and Lime Cooking Extrusion. J. Chem..

[2] Menchaca-Armenta M., Ramírez-Wong B., Torres-Chávez P.I., Quintero-Ramos A., Ledesma-Osuna A.I., Frutos M.J., Gutiérrez-Dorado R., Campas-Baypoli O.N., Morales-Rosas I. (2020). Effect of extrusion conditions on the anthocyanin content, functionality, and pasting properties of obtained nixtamalized blue corn flour (*Zea mays* L.) and process optimization. J. Food Sci..

[3] Mojica L., Berhow M., de Mejia E.G. (2017). Black bean anthocyanin-rich extracts as food colorants: Physicochemical stability and antidiabetes potential. Food Chem..

[4] Mora-Rochín S., Gaxiola-Cuevas N., Gutiérrez-Uribe J.A., Carrillo J.M., Milán-Noris E.M., Reyes-Moreno C., Serna-Saldívar S.O., Cuevas-Rodriguez E.O. (2016). Effect of traditional nixtamalization on anthocyanin content and profile in Mexican blue maize (*Zea mays* L.) landraces. LWT.

[5] Damián-Medina K., Salinas-Moreno Y., Milenkovic D., Figueroa-Yáñez L., Marino-Marmolejo E., Higuera-Ciapara I., Vallejo-Cardona A., Lugo-Cervantes E. (2020). In silico analysis of antidiabetic potential of phenolic compounds from blue corn (*Zea mays* L.) and black bean (*Phaseolus vulgaris* L.). Heliyon.

[6] Brennan C., Brennan M., Derbyshire E., Tiwari B.K. (2011). Effects of extrusion on the polyphenols, vitamins and antioxidant activity of foods. Trends Food Sci. Technol..

[7] Smorowska A.J., Żołnierczyk A.K., Nawirska-Olszańska A., Sowiński J., Szumny A. (2021). Nutritional Properties and In Vitro Antidiabetic Activities of Blue and Yellow Corn Extracts: A Comparative Study. J. Food Qual..

[8] Herrera-Sotero M.Y., Cruz-Hernández C.D., Oliart-Ros R.M., Chávez-Servia J.L., Guzmán-Gerónimo R.I., González-Covarrubias V., Cruz-Burgos M., Rodríguez-Dorantes M. (2019). Anthocyanins of Blue Corn and Tortilla Arrest Cell Cycle and Induce Apoptosis on Breast and Prostate Cancer Cells. Nutr. Cancer.

[9] Pyo Y.-H., Lee T.-C., Logendra L., Rosen R.T. (2004). Antioxidant activity and phenolic compounds of Swiss chard (*Beta vulgaris* subspecies *cycla*) extracts. Food Chem..

[10] Gamba M., Raguindin P.F., Asllanaj E., Merlo F., Glisic M., Minder B., Bussler W., Metzger B., Kern H., Muka T. (2020). Bioactive compounds and nutritional composition of Swiss chard (*Beta vulgaris* L. var. *cicla* and *flavescens*): A systematic review. Crit. Rev. Food Sci. Nutr..

[11] Fernández-López J., Botella-Martínez C., De Vera C.N.-R., Sayas-Barberá M.E., Viuda-Martos M., Sánchez-Zapata E., Pérez-Álvarez J.A. (2020). Vegetable Soups and Creams: Raw Materials, Processing, Health Benefits, and Innovation Trends. Plants.

[12] Camacho-Hernández I., Zazueta-Morales J., Gallegos-Infante J., Aguilar-Palazuelos E., Guzmán N.E.R., Cortez R.O.N., Jacobo-Valenzuela N., Gomez-Aldapa C. (2014). Effect of extrusion conditions on physicochemical characteristics and anthocyanin content of blue corn third-generation snacks. CyTA-J. Food.

[13] Escalante-Aburto A., Ramírez-Wong B., Torres-Chávez P.I., López-Cervantes J., Figueroa-Cárdenas J.D.D., Barrón-Hoyos J.M., Morales-Rosas I., Ponce-García N., Gutiérrez-Dorado R. (2014). Obtaining Ready-to-Eat Blue Corn Expanded Snacks with Anthocyanins Using an Extrusion Process and Response Surface Methodology. Molecules.

[14] Pérez-Navarrete C., Cruz-Estrada R.H., Chel-Guerrero L., Betancur-Ancona D. (2006). Caracterización física de extrudidos preparados con mezclas de harinas de maíz QPM *(Zea mays* L.) y fríjol lima (*Phaseolus lunatus* L.). Rev. Mex. Ing. Quim..

[15] Schmid V., Steck J., Mayer-Miebach E., Behsnilian D., Bunzel M., Karbstein H., Emin M. (2021). Extrusion Processing of Pure Chokeberry (*Aronia melanocarpa*) Pomace: Impact on Dietary Fiber Profile and Bioactive Compounds. Foods.

[16] Zhang R., Khan S.A., Chi J., Wei Z., Zhang Y., Deng Y., Liu L., Zhang M. (2018). Different effects of extrusion on the phenolic profiles and antioxidant activity in milled fractions of brown rice. LWT.

[17] Serna-Saldivar S.O., Chuck-Hernandez C. (2018). Food Uses of Lime-Cooked Corn with Emphasis in Tortillas and Snacks. Corn.

[18] Colombo R., Ferron L., Papetti A. (2021). Colored Corn: An Up-Date on Metabolites Extraction, Health Implication, and Potential Use. Molecules.

[19] Francavilla A., Joye I.J. (2020). Anthocyanins in Whole Grain Cereals and Their Potential Effect on Health. Nutrients.

[20] Luna-Vital D.A., Li Q., West L., West M., de Mejia E.G. (2017). Anthocyanin condensed forms do not affect color or chemical stability of purple corn pericarp extracts stored under different pHs. Food Chem..

[21] Patras A., Brunton N., O’Donnell C., Tiwari B. (2010). Effect of thermal processing on anthocyanin stability in foods; mechanisms and kinetics of degradation. Trends Food Sci. Technol..

[22] Qin X., Yuan D., Wang Q., Hu Z., Wu Y., Cai J., Huang Q., Li S., Liu G. (2018). Maillard-Reacted Whey Protein Isolates Enhance Thermal Stability of Anthocyanins over a Wide pH Range. J. Agric. Food Chem..

[23] Wu H.-Y., Yang K.-M., Chiang P.-Y. (2018). Roselle Anthocyanins: Antioxidant Properties and Stability to Heat and pH. Molecules.

[24] Hong H., Netzel M., O’Hare T. (2020). Optimisation of extraction procedure and development of LC–DAD–MS methodology for anthocyanin analysis in anthocyanin-pigmented corn kernels. Food Chem..

[25] Rabanal-Atalaya M., Medina-Hoyos A. (2021). Análisis de antocianinas en el maíz morado (*Zea mays* L.) del Perú y sus propiedades antioxidantes. Rev. Terra Latinoam..

[26] Ursu M., Aprodu I., Milea Ș., Enachi E., Râpeanu G., Bahrim G., Stănciuc N. (2020). Thermal Degradation Kinetics of Anthocyanins Extracted from Purple Maize Flour Extract and the Effect of Heating on Selected Biological Functionality. Foods.

[27] Delgado-Nieblas C., Ruiz-Beltrán K., Sánchez-Lizárraga J., Zazueta-Morales J.D.J., Aguilar-Palazuelos E., Carrillo-López A., Camacho-Hernández I.L., Quintero-Ramos A. (2019). Effect of extrusion on physicochemical, nutritional and antioxidant properties of breakfast cereals produced from bran and dehydrated naranjita pomace. CyTA-J. Food.

[28] Ruiz-Armenta X.A., Zazueta-Morales J.D.J., Delgado-Nieblas C.I., Carrillo-López A., Aguilar-Palazuelos E., Camacho-Hernández I.L. (2019). Effect of the extrusion process and expansion by microwave heating on physicochemical, phytochemical, and antioxidant properties during the production of indirectly expanded snack foods. J. Food Process. Preserv..

[29] Sun Y., Zhang Y., Xu W., Zheng X. (2020). Analysis of the Anthocyanin Degradation in Blue Honeysuckle Berry under Microwave Assisted Foam-Mat Drying. Foods.

[30] Jacques-Fajardo G.E., Prado-Ramírez R., Arriola-Guevara E., Carrillo E.P., Espinosa-Andrews H., Morales G.M.G. (2017). Physical and hydration properties of expanded extrudates from a blue corn, yellow pea and oat bran blend. LWT.

[31] Leyva-Corral J., Quintero-Ramos A., Camacho-Dávila A., Zazueta-Morales J.D.J., Aguilar-Palazuelos E., Ruiz-Gutiérrez M.G., Meléndez-Pizarro C.O., Ruiz-Anchondo T.D.J. (2016). Polyphenolic compound stability and antioxidant capacity of apple pomace in an extruded cereal. LWT.

[32] Escalante-Aburto A., Ramirez-Wong B., Torres-Chávez P., Figueroa-Cárdenas J., López-Cervantes J., Barrón-Hoyos J., Morales-Rosas I. (2013). Effect of extrusion processing parameters on anthocyanin content and physicochemical properties of nixtamalized blue corn expanded extrudates. CyTA-J. Food.

[33] Gomez-Aldapa C., Navarro-Cortez R., Aguilar-Palazuelos E., Zazueta-Morales J., Rosas J.C., Hernandez-Ávila J., Aguirre-Tostado F. (2014). Microstructure of an Extruded Third-Generation Snack Made from a Whole Blue Corn and Corn Starch Mixture. Int. J. Food Process. Technol..

[34] Neder-Suárez D., Quintero-Ramos A., Meléndez-Pizarro C.O., Zazueta-Morales J.D.J., Paraguay-Delgado F., Ruiz-Gutiérrez M.G. (2021). Evaluation of the physicochemical properties of third-generation snacks made from blue corn, black beans, and sweet chard produced by extrusion. LWT.

[35] Sánchez-Madrigal M.Á., Quintero-Ramos A., Amaya-Guerra C.A., Meléndez-Pizarro C.O., Castillo-Hernández S.L., Aguilera-González C.J. (2019). Effect of Agave Fructans as Carrier on the Encapsulation of Blue Corn Anthocyanins by Spray Drying. Foods.

[36] Nayak B., Berrios J.D.J., Powers J.R., Tang J. (2011). Effect of Extrusion on the Antioxidant Capacity and Color Attributes of Expanded Extrudates Prepared from Purple Potato and Yellow Pea Flour Mixes. J. Food Sci..

[37] Zhao M., Luo Y., Li Y., Liu X., Wu J., Liao X., Chen F. (2013). The identification of degradation products and degradation pathway of malvidin-3-glucoside and malvidin-3,5-diglucoside under microwave treatment. Food Chem..

[38] Yue X., Xu Z. (2008). Changes of Anthocyanins, Anthocyanidins, and Antioxidant Activity in Bilberry Extract during Dry Heating. J. Food Sci..

[39] Xu B., Chang S.K.C. (2009). Total Phenolic, Phenolic Acid, Anthocyanin, Flavan-3-ol, and Flavonol Profiles and Antioxidant Properties of Pinto and Black Beans (*Phaseolus vulgaris* L.) as Affected by Thermal Processing. J. Agric. Food Chem..

[40] Teixeira R.F., Benvenutti L., Burin V.M., Gomes T.M., Ferreira S.R.S., Zielinski A.A.F. (2021). An eco-friendly pressure liquid extraction method to recover anthocyanins from broken black bean hulls. Innov. Food Sci. Emerg. Technol..

[41] Wang X., Hansen C., Allen K. (2013). Identification of Anthocyanins Isolated from Black Bean Canning Wastewater by Macroporous Resin Using Optimized Conditions. Food Nutr. Sci..

[42] Ti H., Zhang R., Zhang M., Wei Z., Chi J., Deng Y., Zhang Y. (2015). Effect of extrusion on phytochemical profiles in milled fractions of black rice. Food Chem..

[43] White B.L., Howard L.R., Prior R.L. (2010). Polyphenolic Composition and Antioxidant Capacity of Extruded Cranberry Pomace. J. Agric. Food Chem..

[44] Del Pozo-Insfran D., Saldivar S.O.S., Brenes C.H., Talcott S.T. (2007). Polyphenolics and Antioxidant Capacity of White and Blue Corns Processed into Tortillas and Chips. Cereal Chem. J..

